# Temperature Rise of an Adhesive Particle-Reinforced Polymer during Fatigue Testing

**DOI:** 10.3390/polym15030742

**Published:** 2023-02-01

**Authors:** Andrzej Komorek, Robert Szczepaniak, Paweł Przybyłek, Kacper Komorek, Zenon Komorek, Jan Godzimirski, Andrzej Zbrowski

**Affiliations:** 1 Department of Aviation, Polish Air Force University, 08-530 Dęblin, Poland; 2 Institute of Materials Science and Engineering, Military University of Technology, 00-908 Warsaw, Poland; 3 Department of Mechatronics, Armament and Aerospace, Military University of Technology, 00-908 Warsaw, Poland; 4 Łukasiewicz Research Network—Institute for Sustainable Technologies, ul. K. Pułaskiego 6/10, 26-600 Radom, Poland

**Keywords:** adhesive composition, polymer, temperature, fatigue, epoxy resin

## Abstract

Construction adhesives are usually polymers that have been modified to achieve specific properties so that they can be used under various loading conditions. An attempt was made to estimate the effect of fatigue loading on the temperature of an adhesive material by further physically modifying the basic adhesive composition used in the research. The temperature of the materials during the tests was recorded using a thermal imaging camera and a thermoelectric thermometer. For most materials tested at 20 Hz, an increase in the number of load cycles corresponded to an increase in the temperature of the samples. For a frequency of 30 Hz, after the temperature increased by a certain value, the temperature of the modified samples recorded with the thermal imaging camera decreased. Fatigue loading caused an increase of the temperature of all tested polymeric materials. Observation of the sample during testing with a thermal imaging camera allows a simple identification of the areas with the highest temperature and can be much more useful in practice than recording temperatures with a thermocouple thermometer, as thermocouples need to be properly positioned before testing.

## 1. Introduction

Despite multiple years of widespread use, bonding technology is a relatively new technique for permanently connecting structural components [1]. It allows new and often better conditions for design, manufacture and repair [2]. Adhesive materials are often used in the construction and repair of various structures, including means of land, air and water transport [3,4]. As a result of the numerous advantages of adhesive bonding and the continuous development of adhesive materials, the share of all adhesive bonds among all construction joints is almost 10%.

The mechanical properties of adhesives, which are predominantly polymeric materials, greatly influence the cohesive strength of construction joints using them [5,6]. In order to estimate the mechanical properties of adhesive materials, destructive experimental tests are usually performed. Their findings allow determining the strength characteristics of an examined adhesive composition [7,8].

Many structures are subjected to varying loads during their service life [9,10], which may result in sudden, unexpected failure of the loaded structure at stresses much lower than their ultimate strength [11,12]. Adhesive structures have high resistance to fatigue loading if the adhesive joints are loaded well below their static strength [13]. The fatigue durability of adhesive joints depends upon the properties of the hardened adhesive composition with which the adhesive joint was made [14,15,16]. The strength properties of structural adhesives (polymers) are highly dependent on the temperature at which they are exploited [17]. With the increase of temperature, the Young’s modulus and tensile strength decrease, but the ductility of the polymer increases [18]. Often, even a minor change in temperature causes a significant change in these properties [19]. In the case of variable loads, the very nature of the load may cause the temperature of the adhesive bond joint to rise by 20–30 °C or even more. A change in the properties of a cured joint usually leads to a change in the strength of the whole bonded structure [20,21,22]. The increase in temperature has a significant impact on the fatigue life of adhesives. In [23], the authors observed that the number of fatigue cycles to failure increased eight times for two types of structural adhesives when the test temperature was reduced from 40 to 25 °C. It can be assumed, however, that due to good heat dissipation of the bonded metal parts, the problem of a temperature rise in this type of joints may be insignificant; however, without conducting appropriate tests, it is impossible to estimate an impact of this phenomenon. It should also be noted that although the glue joints usually have a small thickness, justified by their mechanical strength [24,25], sometimes the adhesive layer may be thicker and have a larger volume, which may adversely affect its properties when the temperature rises. In addition, it should be noted that adhesives are also used to join components made of non-metallic materials, which can conduct heat very poorly away from adhesive joints in fatigue-loaded structures. It therefore appears to be important for the assessment of the properties of adhesive joints in fatigue-loaded structures to estimate the effect of varying loads upon the temperature of the polymer material.

This article presents the findings of a study on estimating the effect of the frequency of fatigue load changes upon the temperature of an adhesive material. The basic glue composition designed for testing was modified physically through the addition of particles of various materials. Physical modifications to the base polymer were targeted to determine the effect of different dispersed phase particles on the material temperature during testing.

The temperatures of the samples were recorded using two methods: a thermal imaging camera and a thermoelectric thermometer.

## 2. Materials and Methods

### 2.1. Materials and Methods for Preliminary Research

The authors selected the Polish epoxy resin Epidian 57 (CIECH Sarzyna S.A, Sarzyna, Poland), cured with triethylenetetramine (hardener Z-1 (CIECH Sarzyna S.A, Sarzyna, Poland)) for 7 days at room temperature (20 °C) and a relative humidity of 50%, for the research. The adhesive cured at ambient temperature had a compressive strength of Rc = 62 MPa and a modulus of elasticity of E = 1830 MPa. Five different types of filler particles were used to modify the composition of the basic composition:

- 10% tungsten (T) powder,
- Expancel microballoons (microspheres) in a quantity of 1%,
- layered silicate montmorillonite (MMT) in an amount of 10%,
- Micronal DS5038X PCM in an amount of 5%,
- silicon carbide (SiC) with a grit size of 220 in an amount of 10%.

In all compositions, the percentage content was measured by weight.

The selection of the types of particles modifying the composition of the base adhesive composition was made on the basis of an analysis of the characteristic properties of the materials. 

It was assumed that tungsten in a powder form (particle size 3–4 µm) as a modifier of the Epidian 57/Z-1 adhesive composition would improve the strength properties of such a material [26,27], as well as increase the thermal conductivity of the composition, which would allow better heat dissipation. Micronal is specialized particles with temperature stabilization properties used primarily in building constructions [28]. Micronal contains a latent heat storage material made of a special blend of waxes in the core of the microcapsule (size: approximately 5 μm). When the temperature rises above a certain temperature threshold (around 26 °C), it absorbs the excess heat energy and stores it in a phase transition. When the temperature drops below the temperature threshold, the capsule releases the stored thermal energy again. Expancel microspheres, on the other hand, are particles which, due to their structure, are characterized by poor thermal conductivity [29] (unlike tungsten), which allows the evaluation of this feature on the rate of heat removal from the tested material. Montmorillonite is a material from the group of nano-additives used in ablative composites, i.e., materials that form barriers against the effects of heat flux. This layered silicate formed by hydrothermal transformations of dust or rock is one of the most added additives to organic-inorganic hybrid nanocomposites. The clay mineral montmorillonite is characterized by high water absorption, reactivity toward acids and ion exchange with organic and inorganic cations. It is a crystalline material consisting of tetrahedral structures based on oxygen, aluminum, magnesium, iron, silicon atoms and hydroxyl groups [30]. A montmorillonite plate measures approximately 1nm and is no less than 100–150 nm long. Silicon carbide is a hard material with very good thermal conductivity (similar to tungsten), which can affect the rate of heat removal from the tested polymer. Silicon carbide, with a grain size of 53–75 µm, was used for the needs of the research. Due to the use of different additives, the internal structure of the tested materials (compositions of Epidian 57/Z-1 resin and reinforcing particles) differed in particle size, density, hardness, etc.

**Figure 1:**
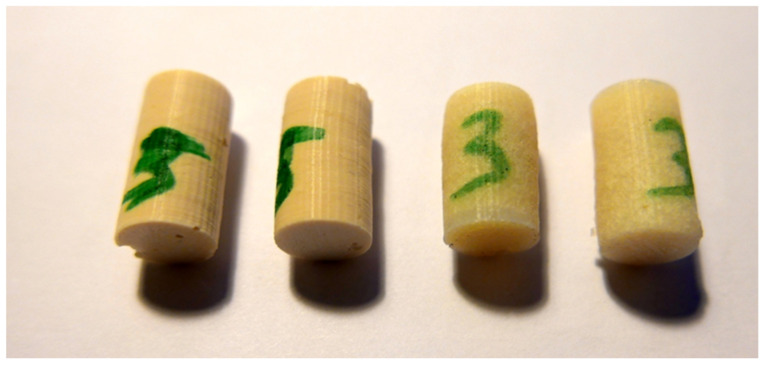
Samples after static tests.

The samples for compressive testing were made by means of a gravity casting method using MM922 silicone. The diameter of the samples equaled 14 mm, whereas the height was 26 mm (Figure 1). The amount of each batch amounted to five. 

### 2.2. Preparation of Specimens for Fatigue Testing

Preparation for the proper tests began with the making thermocouples, whose measuring junctions were placed inside the cast specimens to act as transmitters for the thermoelectric thermometer.

**Figure 2:**
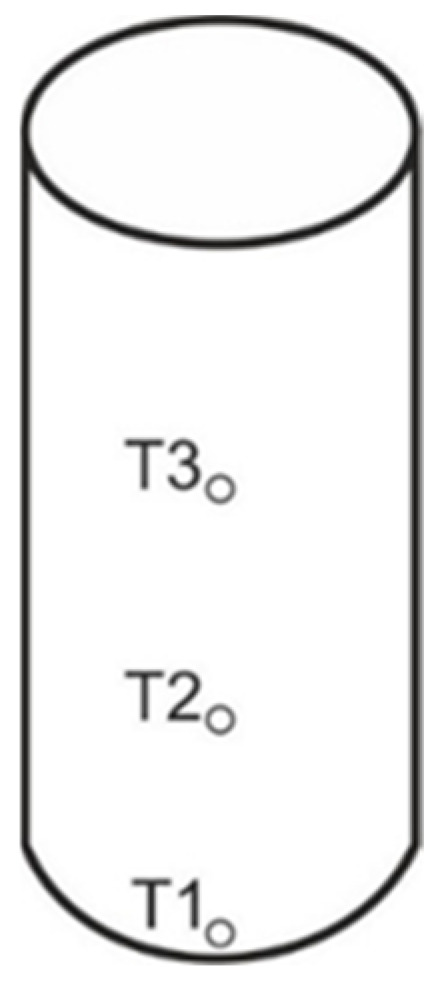
Diagram of the thermocouples placing in the samples (T1—thermocouple no. 1, T2—thermocouple no. 2, T3—thermocouple no. 3).

It was assumed that when using a thermoelectric thermometer, temperature would be determined at three heights: at mid-height, at ¼ height and at the bottom of the sample. The diagram of distributing the thermocouples is shown in Figure 2.

Chromel-alumel sheathed wires (K-type thermocouple) were used as thermoelectrodes to produce thermocouples. Thermocouples with a length of approximately 1 m were prepared for a comfortable measurement in a way that the cable was long enough to plug into the measuring device during the fatigue test.

In order to make the cast polymeric samples, molds were made of molding silicone, the important advantage of which is its very weak adhesion to polymers, making it easy to remove the samples from the mold. Specimen molds were prepared for compression testing, for the determination of the density of the examined polymeric materials and for the determination of the thermophysical properties of the modified polymers.

**Figure 3:**
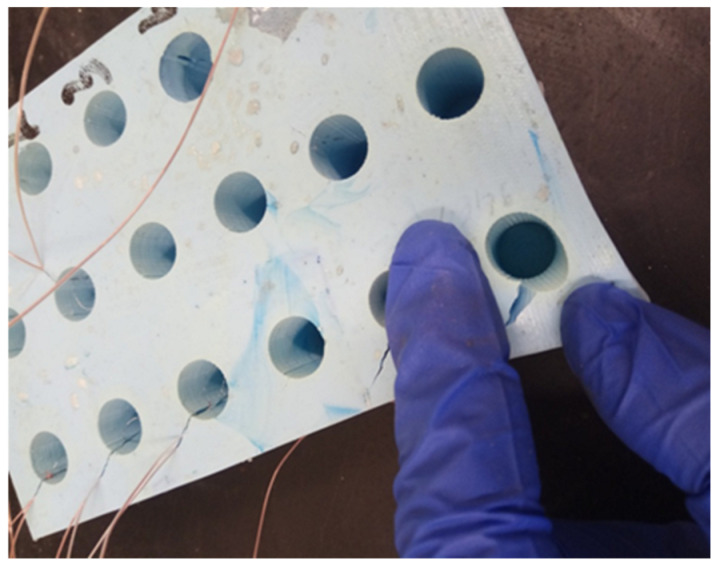
Notches for thermocouples in a silicone form.

The prepared silicone mold allowed for a single casting of 18 compression specimens with a diameter of 11.6 mm and a height of 25 mm. In each of the 18 specimen holes, incisions were made with a bookbinder’s knife (Figure 3) to insert the thermocouples and ensure their attachment during casting of the specimens from the prepared adhesive compositions.

Epidian 57 with hardener Z-1 was used to make the samples. Three specimens of each material were prepared for fatigue testing. The first batch was made from Epidian 57 with a hardener, without additives. Tungsten, montmorillonite, Expancel microballoons, Micronal DS5038X and silicon carbide were added to subsequent samples in the previously specified amounts.

Based on the previous experiments, some compositions were mixed manually with a stirrer and some, due to their form and properties, had to be mixed with an ultrasonic cleaner. The ultrasonic cleaner was used to prepare an adhesive composition with Micronal and montmorillonite. After mixing, each composition was allowed to stand for 5 m, and then the samples were cast in silicone molds. In the mold for the fatigue test specimens, three thermocouples were placed in each of the previously prepared notches before the mixtures were poured. When casting specimens for fatigue testing, specimens for density testing were also made in another mold. After pouring, the samples were left to stand for 7 days at room temperature. After this time, all the specimens were removed from the mold and prepared for fatigue testing by aligning the cylindrical bases of the specimens.

## 3. Research and Discussion

The experimental investigations started with preliminary studies, in which the basic mechanical properties of the epoxy resin Epidian 57/Z1 modified with selected particles were determined. For this purpose, static compressive strength tests were carried out on samples of unmodified and modified resin with a hardener. The results of the tests made it possible to determine the behavior of the compositions produced under load, which will initially make it possible to estimate their suitability as adhesive materials.

Static compressive strength tests were conducted in accordance with EN ISO 604:2006 using a Zwick and Roell Z100 universal static testing machine (Zwick Roell, Ulm, Germany). The research speed was equal to 2 mm/min. 

**Figure 4:**
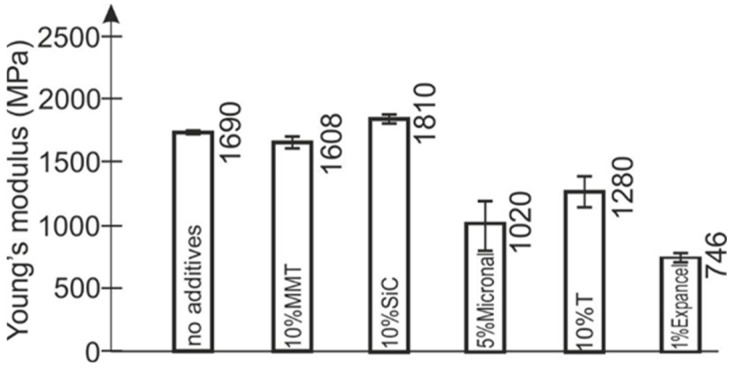
Value of Young’s modulus of the tested materials when compressed.

**Figure 5:**
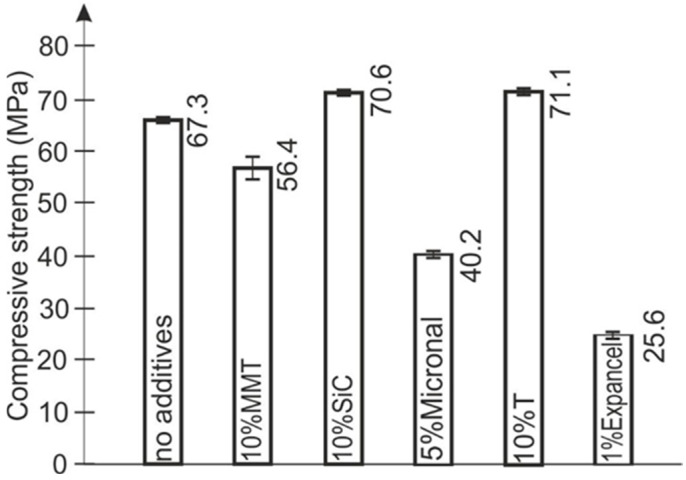
Compressive strength of the tested materials.

At the of the conducted research, the authors determined the Young’s modulus (Figure 4) and the compressive strength (Figure 5) of the tested materials. 

Powder additives, in most cases, caused a decrease in Young’s modulus values, even by more than 50% compared to the unmodified composition (Figure 4). Only the addition of 10% silicon carbide resulted in an increase of the Young’s modulus value.

Epidian 57/Z-1 modified with silicon carbide and tungsten had higher compressive strength than the unmodified resin (Figure 5). The increase in strength had a similar value for both additives (several percent). The highest (more than 60%) decrease in compressive strength was obtained for compositions with 1% addition of Expancel microspheres, which was related to their structure.

### 3.1. Density Testing

**Figure 6:**
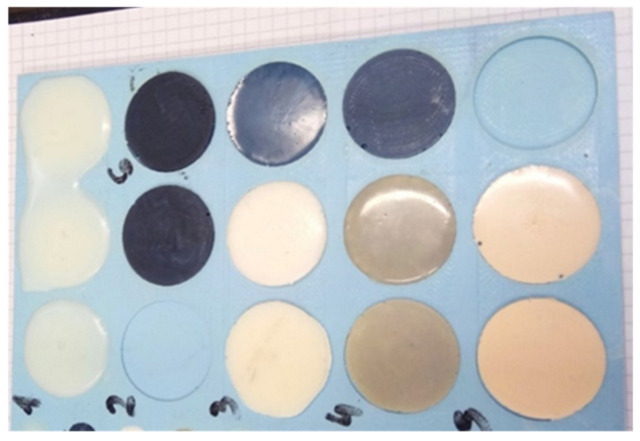
Specimens for density testing.

The density test was carried out using a specialized laboratory balance with an accuracy of 0.00001 g. The cylindrical samples (Figure 6) of 12.7 mm diameter and 2 mm height were tested. The samples were prepared by smoothing and grinding their upper surfaces so that they were flat, without flaws or deformations.

The determined densities of the polymer compositions are presented in Table 1.

The addition of Micronal and Expancel microballoons reduced the density of the Epidian 57/Z-1 composition.

### 3.2. Fatigue Studies

The specimens were loaded with identical unilateral compressive loads at two load change frequencies of 20 Hz and 30 Hz during the tests, which were selected based on the results of [31]. As the test conditions were intended to be as close as possible to those of the adhesive plastics, the sample without any spacers was placed directly between the metal grips of the machine.

The temperature of the specimen was recorded by two methods during the tests as a function of the number of load cycles. The surface temperature of the sample was observed using thermography [32] and recorded using a Flir IQ60 thermal imaging camera (Teledyne FLIR, Wilsonville, OR, USA).

The local temperatures at three points in the specimen were measured using a thermoelectric thermometer with three thermocouples connected to it, the measuring junctions of which were placed inside the specimen during the fabrication process.

During the tests, the specimens were loaded with unilateral sinusoidal compressive loads with an average value of Fm = 2.0 kN and an amplitude of Fa = 1.4 kN for two load change frequencies. The maximum stress in the examined specimens was 32.2 MPa, and it was approximately equal to half of the compressive strength of Epidian 57/Z-1. All the tests lasted up to 20,000 load cycles or until the specimen had been destroyed. The surface temperature of the sample was recorded with a thermal imaging camera every 1000 cycles, whereas the temperature measured with a thermoelectric thermometer was recorded every 1 s. The testing was carried out using an Instron 8501 fatigue machine (Instron, Norwood, MA, USA).

**Figure 7:**
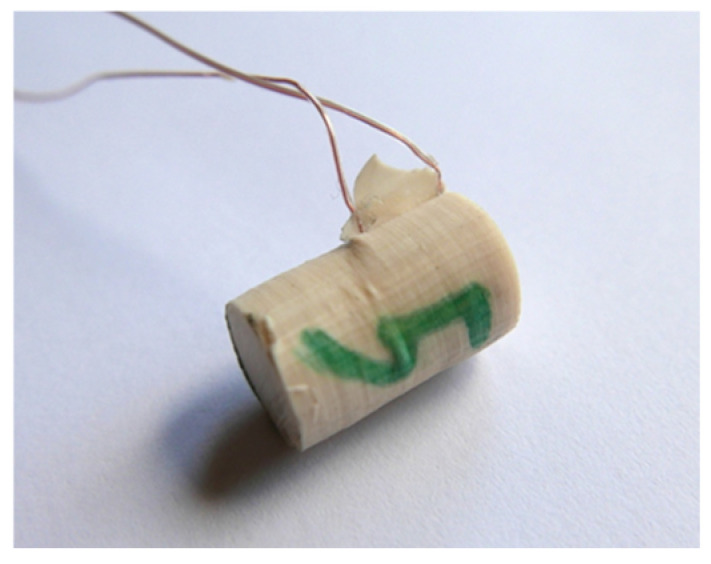
Test sample made with the composition Epidian 57/Z-1 with the 10% addition of ceramic microspheres.

During fatigue testing, it became apparent that the samples were probably too slender and were buckling, and consequently, several of them failed before the end of the specified test cycle. The samples that had failed were made again, yet this time with only two thermocouples each (Figure 7) due to the fact that only small temperature differences between thermocouples T1–T2 and T2–T3 had been found in the first stage of the research. Next, all the samples were cut to the height of 23 mm, which was assumed to be the correct size for testing.

**Figure 8:**
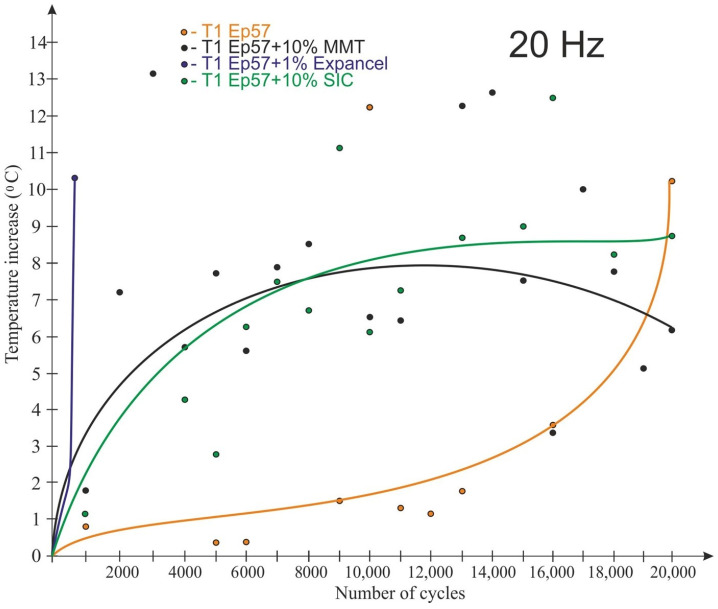
Impact of the number of cycles on the sample temperature at 20 Hz load change frequency—T1 temperature (bottom of the sample).

**Figure 9:**
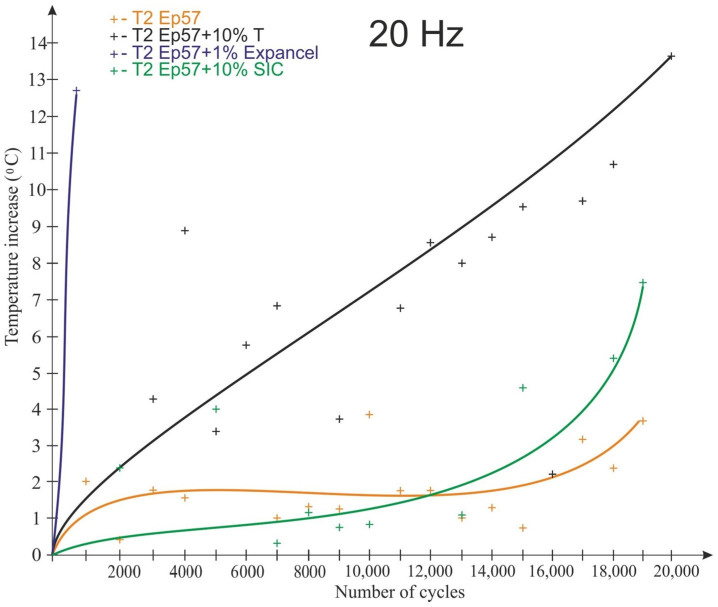
Impact of the number of cycles on the sample temperature at a load change rate of 20 Hz—T2 temperature (1/4 sample height).

**Figure 10:**
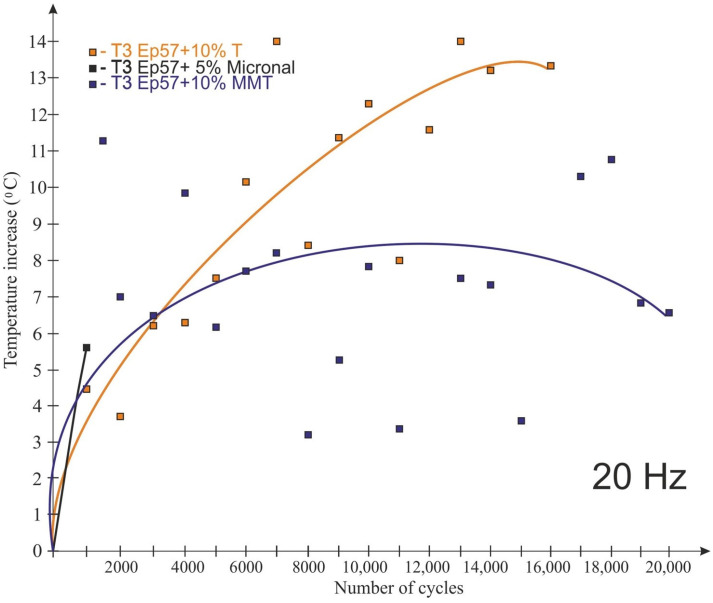
Impact of the number of cycles on the sample temperature at 20 Hz load change frequency—T3 temperature (center of the sample height).

**Figure 11:**
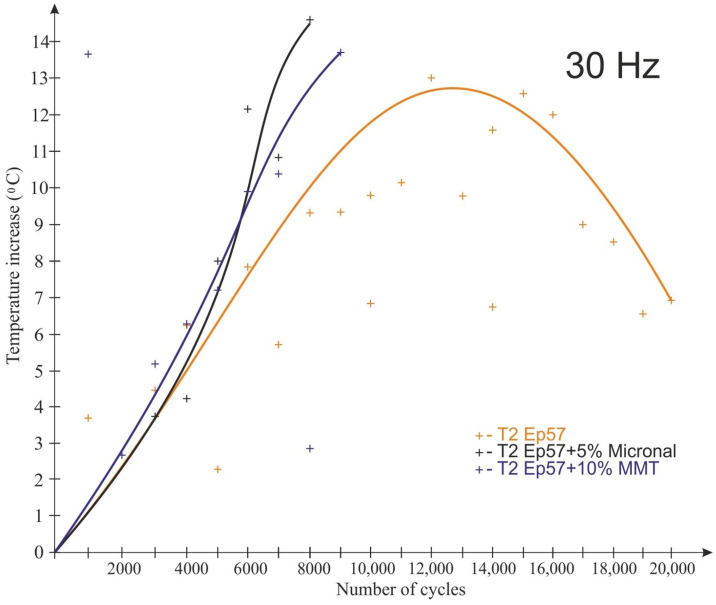
Impact of the number of cycles on the sample temperature at 30 Hz load change frequency—T2 temperature (1/4 sample height).

The graphs shown in Figure 8, Figure 9, Figure 10 and Figure 11 take into account only the temperature measurements made by means of a thermoelectric thermometer. The test results for 20 Hz are shown in Figure 8, Figure 9 and Figure 10 for temperatures T1 (bottom of the sample), T2 (1/4 of the sample’s height) and T3 (middle of the sample’s height), respectively.

As a result of fatigue loading, some of the samples (with Expancel and Micronal microspheres) failed before the end of the assumed test cycle, which was related to the rapid temperature rise. The remaining samples were not destroyed during the test. The lowest temperature rise (about 3.5 °C) at a load variation frequency of 20 Hz was recorded for Epidian 57/Z-1 without additives. It was registered at ¼ of the sample height (Figure 9). The highest temperature rise (about 14 °C) during the tests was observed for the tungsten-modified samples (also at the same height), despite very good heat dissipation of the tungsten particles that had been expected. It seems that in the case of samples with tungsten powder, this behavior could be the result of a low volume proportion of a filler, the consequence of which was the fact that the particles were not in contact with each other, and they did not conduct heat. The rise in temperature in all the samples was non-linear in its nature. It should also be noted that despite great care being taken to properly prepare and mount the thermocouples in the samples, some thermocouples were damaged during the testing.

**Figure 12:**
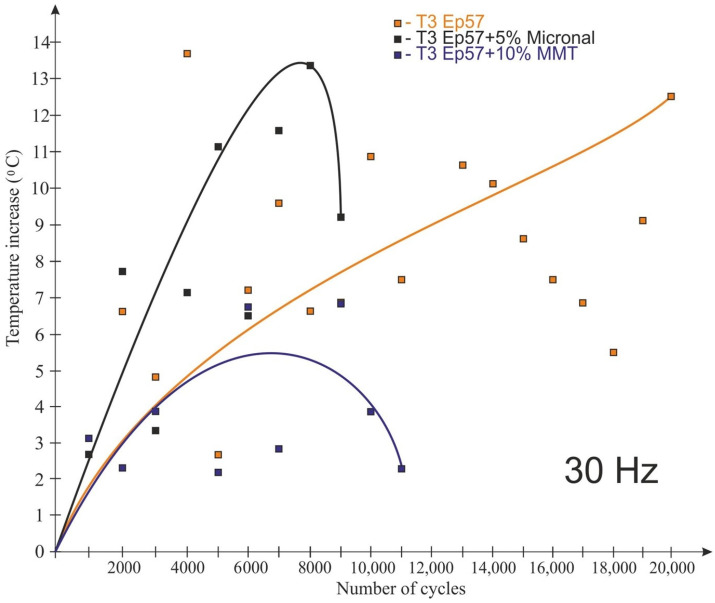
Impact of the number of cycles on the sample temperature at a load change rate of 30 Hz—temperature T3 (center of sample height).

The test results for the frequency of 30 Hz are shown in Figure 11 and Figure 12. The tests conducted at 30 Hz also recorded an increase in the temperature of the examined plastics. Only the samples made of the “pure” composition were not destroyed during the test. Unlike for the 20 Hz tests, the Micronal-modified material had the highest temperature rise, with the highest temperature rise recorded in the ¼ height of the sample (14.4 °C) (Figure 11). In addition, for this material, there was the fastest temperature rise after the start of the test (in the middle of the sample). By far, the lowest temperature rise (T3—in the middle of the sample height) during the test was recorded for the material with the 10% MMT addition, but the sample failed after 11,000 cycles.

**Figure 13:**
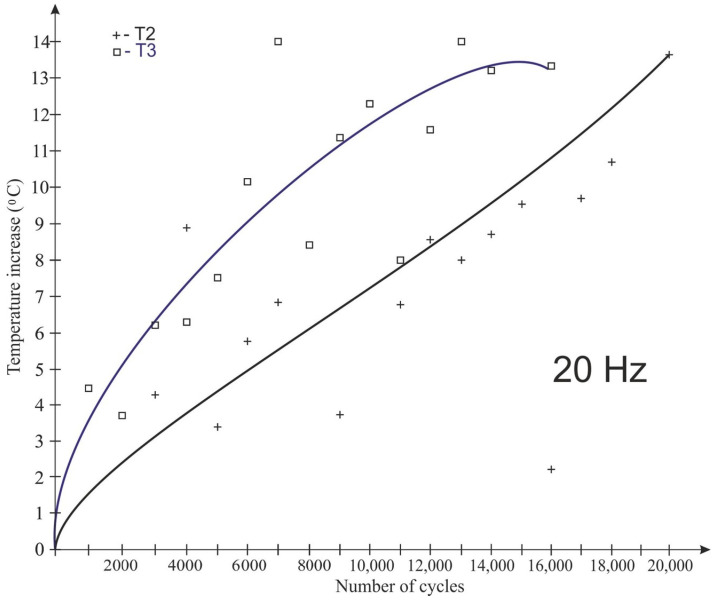
Effect of the frequency of impact load change and the number of cycles upon the temperature of tungsten-modified samples in quantities 10%.

The temperature values recorded at ¼ height and mid-height were also compared for samples made of the same material (Figure 13) at a load change frequency of 20 Hz. Throughout the test, the temperature at ¼ of the sample height was lower than the temperature at the center of the sample height.

**Figure 14:**
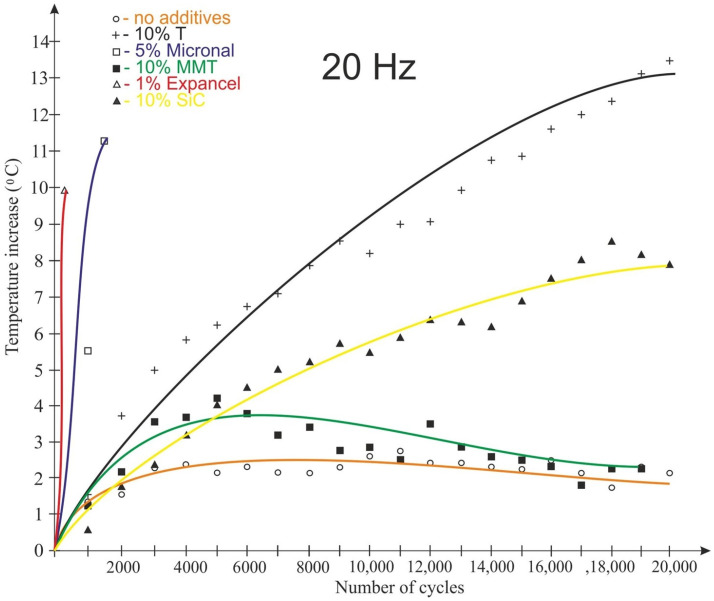
Impact of the number of cycles on the temperature recorded in the center of the samples using a Flir iQ60 thermal imaging camera at a load variation frequency of 20 Hz.

**Figure 15:**
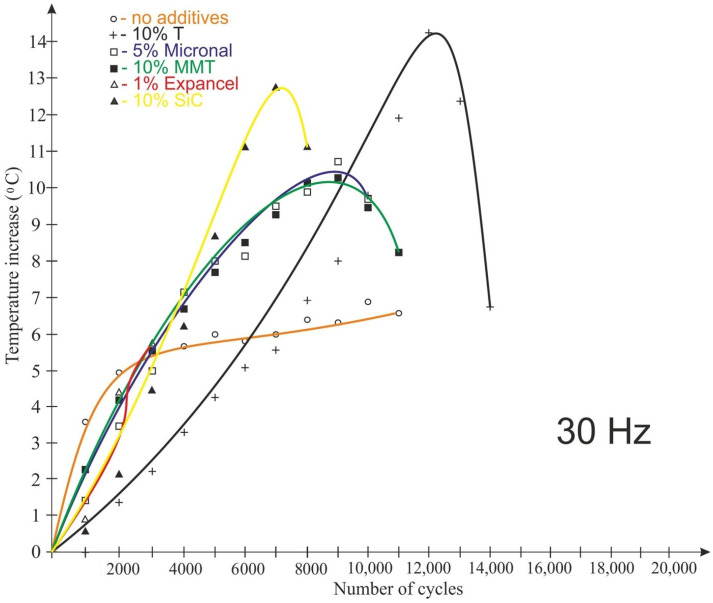
Impact of the number of cycles on the temperature recorded in the center of the samples using a Flir iQ60 thermal imaging camera at a load variation frequency of 30 Hz.

The temperature of the samples read from the thermal imaging camera images is shown in Figure 14 and Figure 15. The temperature observations were made at the center of the sample. For a load variation frequency of 20 Hz, the lowest temperature rise was recorded for samples made from Epidian 57/Z-1 without additives. The temperature of this material increased by 2.5 °C. The highest temperature change in the observation place was recorded for the adhesive composition modified with tungsten powder; in this case, the temperature of the plastic increased by over 13 °C, i.e., similarly to the temperature recorded using a thermoelectric thermometer. The nature of the temperature change recorded using the thermal imaging camera varied between the samples. For samples with Microballoons and Micronal additives, the temperature increase was very rapid and linear (by 10–11 °C) until destruction before reaching 2000 cycles. For samples modified with tungsten and silicon carbide, the temperature rise was constant but non-linear and with a smaller gradient. However, in the case of the Epidian 57/Z-1 composition without additives and the composition with the addition of MMT, after the temperature rise in the initial period of testing (6000–8000 cycles), there was a decrease in the observed temperature until the end of the testing cycle.

**Figure 16:**
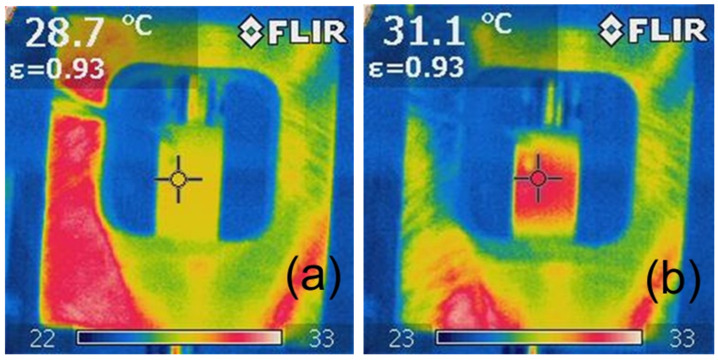
Exemplary thermographic images of specimens during testing of Epidian 57/Z-1 without reinforcement after (**a**) 0 cycles (20 Hz), (**b**) 10,000 cycles (20 Hz).

**Figure 17:**
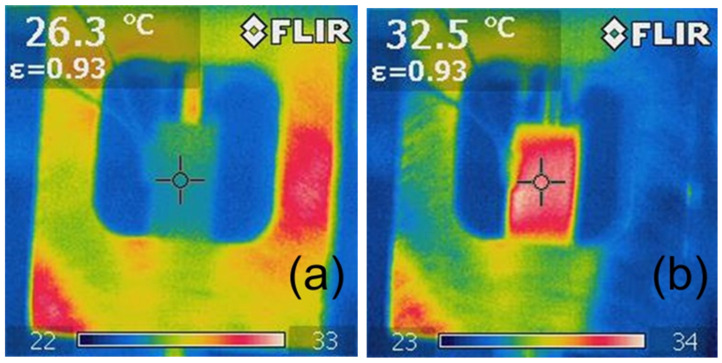
Exemplary thermographic images of samples during testing of Epidian 57/Z-1 +10% SiC resin after (**a**) 0 cycles (20 Hz), (**b**) 15,000 cycles (20 Hz).

**Figure 18:**
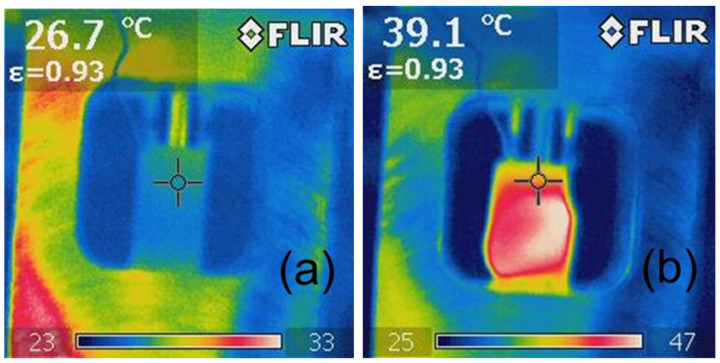
Exemplary thermographic images of samples during testing of Epidian 57/Z-1 +10% W resin after (**a**) 0 cycles (30 Hz), (**b**) 14,000 cycles (30 Hz).

The analysis of the photographic documentation of the tests, showing the surface temperature of the sample during the fatigue test, indicates that the sample reached its highest temperature in the middle of its height (Figure 16, Figure 17 and Figure 18), which is related to the rapid removal of heat from the sample by the metal grips. With taller specimens used in the first stage of testing, the temperature may have had a different distribution, with the highest values shifted toward the grips. Furthermore, it can also be noted that a local increase in the temperature of the sample during testing indicates the location of the failure, which can be used during the performance evaluation of the adhesive bond.

The example thermograms of samples without additives shown in Figure 16 show that the sample areas near the handles had a similar temperature at the beginning of the test and after 10,000 fatigue cycles.

Sample thermograms of samples with additives shown in Figure 17b and Figure 18b indicate that the areas with higher temperature covered almost the entire volume of the samples, and the temperature differences before the tests and after several thousand fatigue cycles were higher than in the case of samples without any additives.

## 4. Conclusions

As a result of fatigue loading, the temperature of all tested polymeric materials increased. The addition of any physical modifier resulted in a higher temperature change compared to the unmodified Epidian 57/Z-1 composition during cyclic compression tests, which is worth paying attention to when we intended to physically modify polymers loaded with fatigue during operation. The reason for this may be the occurrence of friction between the modifier particles and the resin.

In fatigue testing, the dissipation of heat by metal grips from the contacting sample surfaces limits the temperature rise in areas close to these surfaces, so in typical thin-bond adhesive joints, the problem of destructive temperature rise is unlikely. This problem may be important in the case of the adhesive bonding of polymer composites.

For most materials tested at 20 Hz, an increase in the number of load cycles corresponded to an increase in the temperature of the sample. For a frequency of 30 Hz, another observation was made; namely, after the temperature increased by a certain value, the temperature of the modified samples recorded with a thermal imaging camera decreased.

The materials with Micronal and Expancel microsphere additives were destroyed by the rapid temperature rise before the full 20,000-cycle test plan was completed. Lower fatigue loads should be used for this type of material. In order to define universal test conditions for all materials, these conditions should be defined for materials that fail prematurely by performing additional experimental tests.

When conducting compression fatigue tests on cylindrical samples made from polymeric material, care must be taken to ensure that the ratio of specimen height to diameter does not exceed 2:1.

Observation of the sample during testing with a thermal imaging camera allows a simple identification of the areas with the highest temperature and can be much more useful in practice than recording temperatures with a thermocouple thermometer, as thermocouples need to be properly positioned before testing. 

Placing more than two thermocouples in test samples of the dimensions used is not justified due to small temperature differences. 

The authors see the need to investigate polymer composites with different types of reinforcements in the aspect of heating of fatigue-loaded composite materials.

## Figures and Tables

**Table 1:** Density of the tested polymers.

Polymer	Epidian 57 + Z1					
Additive	-	Tungsten	Micronal	Montmorillonite	Mcroballons	SiC
Density (g/cm^3^)	1.157	1.281	1.012	1.218	0.887	1.236

## Data Availability

The raw/processed data required to reproduce these findings cannot be shared at this time due to technical or time limitations.
